# Effect of *Pseudomonas aeruginosa* on Corrosion Behavior of X65 Carbon Steel

**DOI:** 10.3390/ma17102428

**Published:** 2024-05-17

**Authors:** Zixuan Shao, Ruiqi Guo, Jianhua Tang, Xin Zhang

**Affiliations:** 1 School of Materials Science and Engineering, Tianjin University of Technology, Tianjin 300384, China; shaozixuan1229@163.com (Z.S.); rqi477@163.com (R.G.); 2 China National Offshore Oil Corporation, Beijing 100010, China; tangjianhua728@126.com

**Keywords:** microbiologically influenced corrosion, *Pseudomonas aeruginosa*, X65 pipeline steel, pyocyanin

## Abstract

X65 pipeline steel is widely used in the field of offshore oil and gas exploitation due to its excellent performance. However, due to the complex environment in the ocean, X65 pipeline steel is faced with a great risk of microbial corrosion failure. Therefore, it is of great significance to study the corrosion mechanism of X65 pipeline steel by microorganisms. In this paper, the corrosion effect of *Pseudomonas aeruginosa* (*P. aeruginosa*) secreting phenazine compounds on X65 pipeline steel was studied by the weight loss method, biofilm scanning electron microscopy analysis, surface corrosion morphology observation, electrochemical testing and medium pH test corrosion products. The results showed that the inoculation of *P. aeruginosa* accelerated the corrosion of X65 steel. After knocking out the *phzM* and *phzS* genes that regulate the synthesis of PYO, *P. aeruginosa* can still produce biofilms on the surface of X65 steel consistent with the morphology of wild-type *P. aeruginosa*, but the corrosion of X65 steel is significantly reduced. It is proved that PYO plays an important role in the corrosion process of *P. aeruginosa* on steel.

## 1. Introduction

With the rapid development of the economy, China is facing a serious energy shortage, so gradually, focus on the ocean, China’s long coastline, rich in marine resources [1], the realization of the stable development of the marine environment and safe production have become the key to solving the energy problem. The construction of major marine projects such as marine oil and gas pipelines, large offshore platforms, large ships and other major marine projects has led to the rapid development of steel materials for marine engineering [2]. Offshore steel needs to meet the criteria of high strength, good corrosion resistance and good weldability. Carbon steel and stainless steel have been proven by a large number of studies to be able to possess these characteristics and have been put into most offshore applications, such as X65, X70, X80, SS304, SS316L and so on. X65 pipeline steel, as a kind of steel used in marine engineering, has excellent properties, including high strength, high toughness, good weldability, excellent molding and resistance to corrosion and cracking, as well as low production costs, and has been widely used in the oil and gas industry [3,4,5]. However, X65 pipeline steel is prone to serious corrosion in harsh seawater environments, especially the corrosion problem of microorganisms on it [6]. A large number of microorganisms, including bacteria, fungi, algae and lichens, are present in the marine environment, and the surfaces of metallic materials exposed to seawater environments may be rapidly colonized by microorganisms, leading to complex corrosion problems. In 2009, biocatalytic cathodic sulfate reduction (BCSR) was proposed by Gu and Xu et al. [7,8,9,10]. The reduction of cathodic sulfate directly consumes the electrons released from the dissolution of anodic metal materials and accelerates the corrosion of metals [11]. Gu et al. further introduced the extracellular electron transfer (EET) process in microbial fuel cells into microbial corrosion and further explained the mechanism of microbial accelerated corrosion of metals from the bioenergetics point of view.

*P. aeruginosa* dominates the marine environment and readily forms biofilms on metal surfaces. Studies have confirmed that *P. aeruginosa* is involved in the corrosion process of soft steel, stainless steel and aluminum in marine habitats [12,13] and even accelerates the corrosion of duplex and super duplex stainless steels. The unique heterocyclic aromatic ring structure of *P. aeruginosa* accepts electrons and mediates extracellular electron transfer in bacteria by utilizing its secreted phenoxazines as electron carriers to exchange electrons with the outside world [14,15]. A growing number of studies have shown that *P. aeruginosa* is one of the most important bacteria causing marine corrosion and economic and environmental losses [16]. *P. aeruginosa* produces a lot of metabolites during its growth process, and one of the secondary metabolites produced is phenazines, and among all the phenazines, one of the most important ones is chlorophyllin (PYO). Phenazines are synthesized under the control of a series of *phz* genes [17]. It has been shown that in *P. aeruginosa*, phenazine compounds are synthesized by gene clusters, and in this bacterium, there are a total of two gene clusters, each of which is composed of seven genes: phzA1B1C1D1E1F1G1 and phzA2B2C2D2E2F2G2 [18]. These two gene clusters are involved in the synthesis of phenazine-1-carboxylic acid (PCA). There are also three genes in *P. aeruginosa*, *phzH*, *phzM*, and *phzS*, which are involved in the conversion of PCA to other phenazines [19,20,21]. PCA is converted to phenazine-1-carboxamide (PCN) by the action of *PhzH*, and phenazine-1-hydroxyl (1-OH-PHZ) by the action of *PhzS*. The conversion of PCA to phenazine-1-carboxamide (PCN) and phenazine-1-hydroxyl (1-OH-PHZ) occurs by the action of the two phenazine modifier genes encoded by the enzymes *PhzM* and *PhzS* to PYO [22].

Of all the phenazines produced by *P. aeruginosa*, PYO is the most abundant. The activity of PYO is flexible and can freely travel on both sides of the biological membrane, and PYO can also accept and give electrons, as it has a certain redox capacity [23]. Under aerobic conditions, the main electron acceptor of PYO is molecular oxygen. The colorless reduced form of PYO gives an electron to O_2_, which produces a superoxide anion and turns into the blue oxidized form. PYO can also accept electrons produced by the respiratory chain of bacteria and then transfer the electrons to an electron acceptor far away from the bacterial cell and, in this way, allow *P. aeruginosa* to continue to survive in anaerobic conditions.

In this paper, the effect of the gene-regulated endogenous electron mediator PYO on steel MICs was investigated by systematically studying the corrosion behavior of *PhzM* and *PhzS* knockout (M+S) wild-type genes in seawater. Their ability to form biofilms was also analyzed after gene modification. In addition, in this study, the mechanism of EET-MIC action of *P. aeruginosa* on X65 was investigated using electrochemical methods and surface analysis techniques.

## 2. Materials and Methods

### 2.1. Materials

X65 steel specimens (10 mm × 10 mm × 3 mm) provided by China National Offshore Oil Corporation Limited were used for all tests, and the chemical composition of X65 steel is shown in Table 1. For electrochemical tests, the coupons were sealed with epoxy resin, and only one working face, 1 cm^2^ in area, was exposed. All the coupons were sequentially abraded from 400 to 2000 grit and further polished with polishing compound, followed by an ultrasonic cleaning in anhydrous ethanol. Before the tests, coupons were sterilized under UV irradiation for 1 h.

### 2.2. Bacteria and Culture

*P. aeruginosa* (MCCC 1A00099) used in this experiment was purchased from the China Marine Microbial Strain Collection and Management Center, stored in a −80 °C cryogenic refrigerator after encapsulation with glycerol, and first resuscitated in 2216E medium at a controlled temperature of 30 °C before use. The solution used in the immersion experiment was 2216E medium. The composition of the medium was as follows: peptone 5.0 g/L, yeast dipping powder 1.0 g/L, ferric citrate 0.1 g/L, sodium chloride 19.45 g/L, magnesium chloride 5.98 g/L, sodium sulfate 3.24 g/L, calcium chloride 1.8 g/L, potassium chloride 0.55 g/L, sodium carbonate 0.16g/L, potassium bromide 0.08 g/L, strontium chloride 0.034 g/L, boric acid 0.0022 g/L, sodium silicate 0.004 g/L, sodium fluoride 0.0024 g/L, ammonium nitrate 0.0016 g/L, and disodium hydrogen phosphate 0.008 g/L. The chemical reagents used were of analytical purity, and the solvent was deionized water. The vessels, tweezers and liquid medium used in the experiment were autoclaved at 121 °C for 20 min before the experiment, and then they were transferred to the UV lamp for 1 h.

### 2.3. Immersion Experiment

The immersion test was carried out in 50 mL anaerobic vials. Each anaerobic vial was filled with sterilized medium (50 mL), and 1 sample was placed at the bottom of each anaerobic vial, with the working surface placed face up. After inoculation of 1 mL of *P. aeruginosa* solution in the anaerobic vials, the vials were incubated anaerobically at 30 °C for 1, 3, 7, and 14 days, and a sterile group was set up as a control experiment. These were performed on an ultra-clean bench.

### 2.4. Weightlessness Testing and Surface Analysis

**Figure 1:**
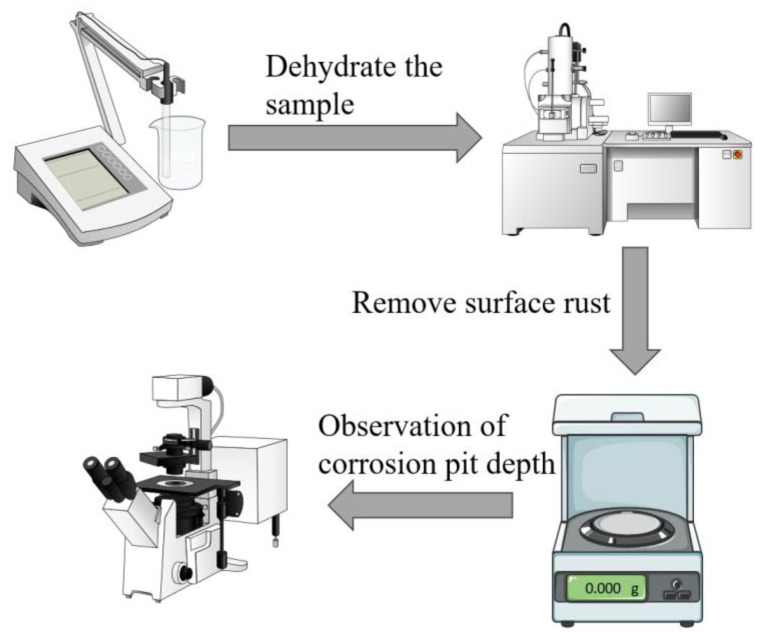
Experimental flowchart for weightlessness testing and surface analysis.

Prior to the immersion test, the specimens were numbered and weighed on an analytical balance. Three parallel specimens were prepared for each set of experiments to ensure the accuracy of the results and to exclude reproducibility. The specimens were removed for dehydration after 1, 3, 7 and 14 days. After the samples were treated with different durations of submergence, 20 mL of the solution was poured from the remaining solution in a dispensing flask. The pH meter (Model PHS-3CB) was calibrated prior to each use, and then the pH meter was used to measure and record the pH of the solution. Figure 1 shows the flowchart of the weightlessness test and surface analysis experiments. The specific steps are as follows:

(1) After the samples were removed from different cycles of immersion, the samples were rinsed with phosphate-buffered saline (PBS) to remove the floating *P. aeruginosa* cells and impurities on the sample surface. The samples were then immersed in 2.5% glutaraldehyde solution for 24 h to immobilize the formed biofilm on the specimen surface, followed by gradual dehydration with ethanol solutions at 50%, 60%, 70%, 80%, 90% and 100% concentrations. To improve the electrical conductivity of the specimen surface, a gold film was sprayed on the surface of the specimen, and then the biofilm was observed by field emission scanning electron microscope (SEM, Quanta FEG 250, FEI, Hillsborough, OR, USA).
(2) Nitric acid, hexamethylenetetramine and deionized water were used to prepare a rust remover to remove biofilm and corrosion products from the surface of X65 steel. Then the samples were washed with deionized water and anhydrous ethanol, and finally, the samples were dried. The weight of the dried samples was weighed and recorded.
(3) The diameter, depth and number of corrosion pits on the surface of the specimens were measured using a confocal laser scanning microscope (CLSM, OLS5000, Olympus, Tokyo, Japan).

### 2.5. Electrochemical Tests

The electrochemical experiments were carried out using an electrochemical workstation (CHI660E, CH Instruments, Austin, TX, USA), and the tests were performed using a three-electrode system, with the working electrode being X65 carbon steel sealed with epoxy resin, the counter electrode being a graphite electrode, and the reference electrode being a saturated calomel electrode. Open circuit potential (OCP) was measured first, and the test time was 600 s. Then electrochemical impedance spectroscopy (EIS) was carried out, and the frequency range of the test was 100,000 Hz~0.01 Hz, and the amplitude of sinusoidal disturbance was 0.005 V. Each test was carried out by the three-electrode system. Each set of experiments was tested three times to ensure reproducibility. At the end of the immersion cycle, the samples were tested for kinetic potential polarization curves at a scan rate of 0.166 mV/s, with a measurement range of −0.5 V~2 V (vs. E_OCP_), and a scan rate of 0.5 mV/s. The corrosion current densities were obtained by extrapolation from the Tafel zone. The data were processed using ZSimpWin software (Version 3.50) to establish an equivalent circuit model.

## 3. Results

### 3.1. Weightlessness Analysis

**Figure 2:**
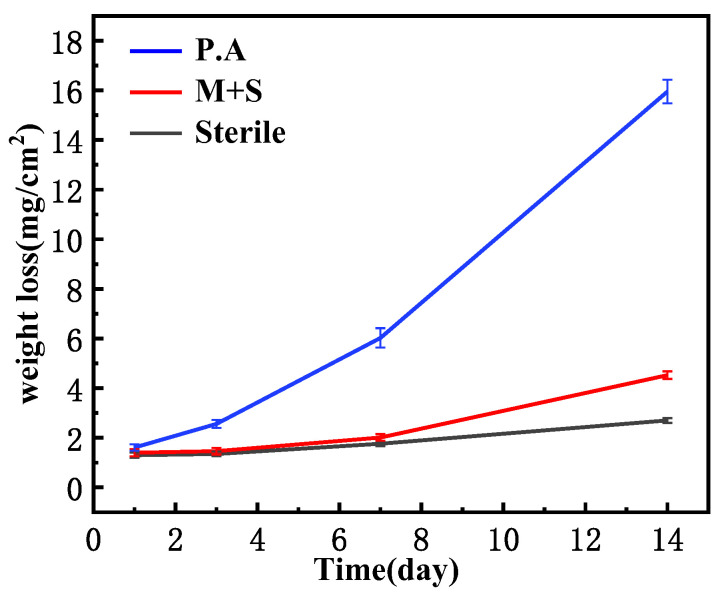
Weight loss results of X65 steel after immersion in different medium for 1, 3, 7 and 14 days.

Figure 2 shows the resulting weight loss of X65 tubular steel immersed in wild type *P. aeruginosa* and knockout *P. aeruginos* conditions for 1, 3, 7 and 14 days. The data results were obtained by averaging three parallel sets of samples. As can be seen from the figure, with the increase of immersion time, the weight loss of immersed in wild-type *P. aeruginosa* medium was more obvious, and the increase of weight loss was more obvious after 14 days, while the weight loss after immersion in the knockout medium was slightly increased compared with sterile, which indicated that the knockout *P. aeruginosa* attenuated corrosion of X65 pipeline steel. Therefore, knockdown of the two genes, *phzM* and *phzS*, which regulate the synthesis of PYO, caused *P. aeruginosa* to weaken the corrosion of X65 pipeline steel.

### 3.2. Surface Morphology

**Figure 3:**
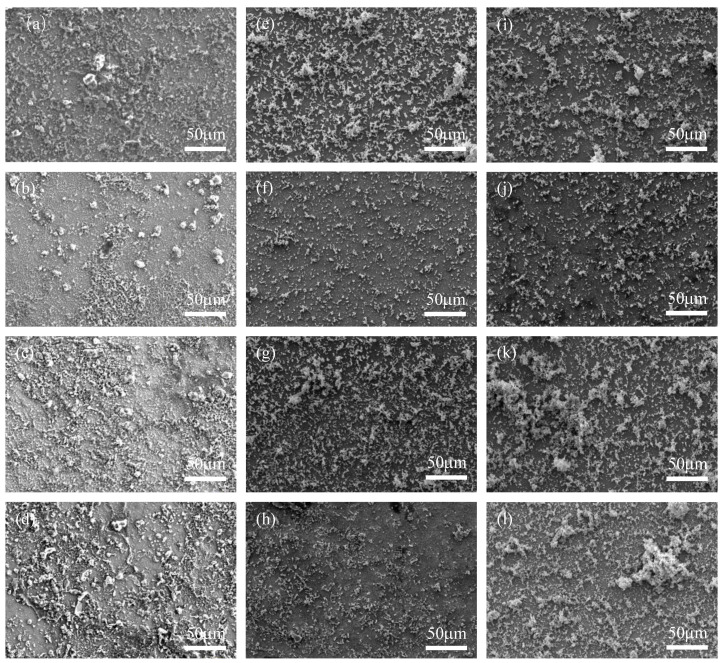
SEM plots of X65 after 1, 3, 7, and 14 days of immersion in sterile medium (**a**–**d**) and medium containing wild-type *P. aeruginosa* (**e**–**h**) and knockout *P. aeruginosa* (**i**–**l**).

Figure 3 shows SEM images of X65 pipeline steel after 1, 3, 7, and 14 days of immersion in medium containing wild-type and knockout *P. aeruginosa*. It can be clearly seen that the steel surface under bacterial conditions is covered with biofilms composed of rod-shaped microorganisms, which are distributed in clusters on the surface of the X65 steel, while the steel surface under aseptic conditions shows corrosion products of different sizes and shapes, which are relatively homogeneous, suggesting that the difference in the number of days of immersion in this condition does not significantly increase or decrease the corrosion rate of the steel. From the morphology of the biofilm, the biofilm produced by the knockout type and the wild type had the same morphology, indicating that the knockout of the *phzM* and *phzS* genes did not have a particularly large effect on the biofilm produced by *P. aeruginosa* on the surface of X65 pipeline steel. However, the amount of bacterial growth still varied depending on the immersion time. Corrosion products produced by both wild-type and knockout *P. aeruginosa* on the X65 steel surface decreased after 3 days of immersion and increased after 7 days of immersion. However, after 14 days of immersion, the corrosion products produced by wild-type *P. aeruginosa* decreased again, while the corrosion products produced by knockout *P. aeruginosa* still increased.

### 3.3. Electrochemical Analysis

**Figure 4:**
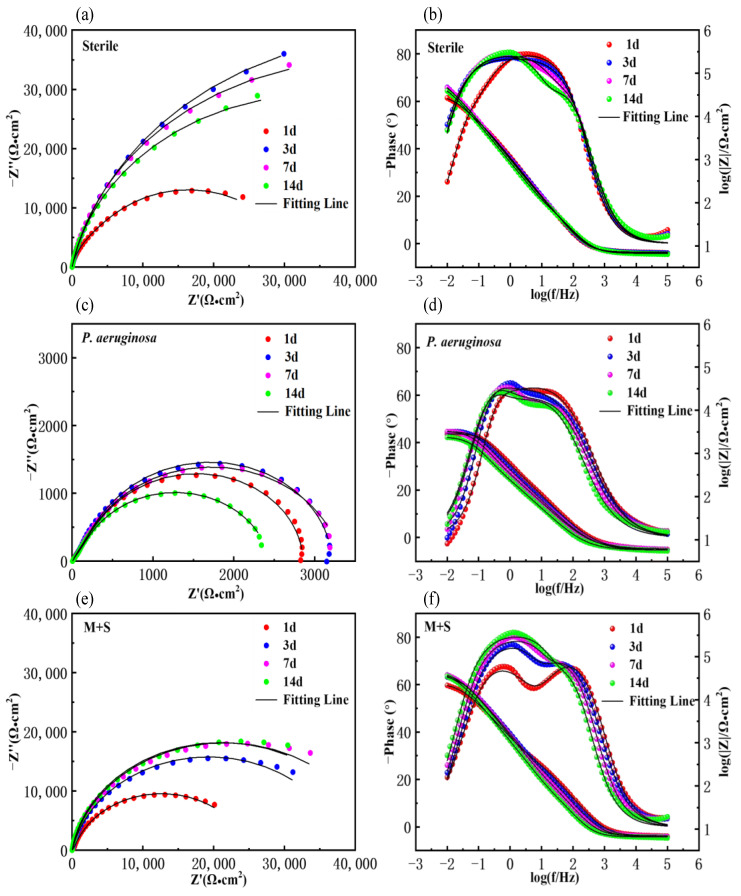
Nyquist and Bode plots of X65 tubular steel in sterile (**a**,**b**), anaerobic medium containing wild-type *P. aeruginosa* (**c**,**d**) and knockout *P. aeruginosa* (**e**,**f**).

Figure 4 shows the Nyquist and Bode plots of X65 pipeline steel after 1, 3, 7, and 14 days of immersion in sterile, wild-type-containing and knockout-containing *P. aeruginosa* medium. As can be seen from the Nyquist plots, the diameters of the Nyquist rings of the medium with bacteria were all smaller than those of the sterile medium, indicating that the presence of microorganisms accelerated the corrosion of the steel, whereas the diameters of the Nyquist rings in the medium inoculated with the knockout type of the genes were much larger than those of the samples inoculated with the wild type, which indicates a significant decrease in the corrosion capacity of the biofilm of the knocked-out *P. aeruginosa* from the knockout of the *phzM* and *phzS* genes, and thus the corrosion of X65 steel was attenuated. Correspondingly, the Bode plots show that the impedance modulus in the low-frequency region (|Z|_0.01 Hz_) exhibited consistent variation patterns as capacitive loops. The larger diameter of a capacitive loop and higher |Z|_0.01 Hz_ value are commonly used as indicators of better corrosion resistance. In the Bode plots, the impedance modulus in the low-frequency region of the X65 steel samples in the medium inoculated with the knockout *P. aeruginosa* was significantly increased. Under aseptic conditions, the diameter of the Nyquist rings increased and then decreased, with the largest diameter at day 3, indicating that the X65 steel was most resistant to corrosion at day 3 and stabilized thereafter. The Nyquist plots in the wild-type medium showed that the diameter of the semicircular arcs gradually increased after 1 and 3 days of immersion, and then decreased after 7 and 14 days of immersion, and the diameter was the largest at day 3, indicating that the corrosion of X65 steel by *P. aeruginosa* was the weakest at day 3. The Nyquist plot in the knockout medium shows that the diameter of the semicircular arc gradually increased, indicating that the corrosion of X65 steel by *P. aeruginosa* knockout was gradually weakened.

**Figure 5:**
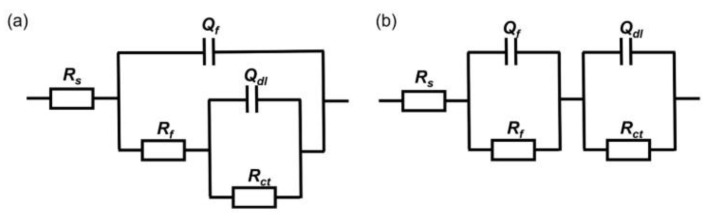
The equivalent electrical circuit used to fit the EIS spectra of coupons immersed in the different media for (**a**) 1 day, 3 days, (**b**) 7days and 14 days.

Figure 5 shows the equivalent circuit fitted to the measured impedance data, where R*_S_* represents the electrolyte resistance, R*_f_* and Q*_f_* represent the resistance and capacitance of the biofilm, and R*_ct_* and Q*_dl_* represent the charge transfer resistance and double-layer capacitance, respectively. In the fitting process, the constant phase element (CPE) is used to reflect the dispersive behavior of the physical phenomena and is defined as follows:

(1) $$Z\left(\omega \right)=Z_{0}-(i\omega {)}^{-n}$$

where *Z*_0_ is the scale factor, *ω* is the angular frequency, and n is the CPE index corresponding to the phase shift. The fitting results are shown in Table 2, where the R*_ct_* value of the medium inoculated with wild-type *P. aeruginosa* was the largest, the lowest corrosion rate, at the 3rd day of immersion. The R*_ct_* value decreased from 3.5 × 10^2^ ± 8.3 Ω∙cm^2^ to 1.7 × 10^2^ ± 8.9 Ω∙cm^2^ after 14 days of immersion and was much lower than that of the other two groups; the corrosion rate was much higher. The R*_ct_* value of the medium inoculated with knockout *P. aeruginosa* gradually increased, indicating that the corrosion rate was gradually decreasing. After 14 days in all of being immersed, the R*_ct_* values of the inoculated knockout *P. aeruginosa* medium (3.8 × 10^4^ ± 2.4 × 10^4^ Ω∙cm^2^) were higher than those of the inoculated wild type (1.7 × 10^2^ ± 8.9 Ω∙cm^2^), which were close to the sterile medium (6.2 × 10^4^ ± 8.3 × 10^2^ Ω∙cm^2^), indicating that the gene knockout weakened the corrosion of X65 steel by *P. aeruginosa* and reduced the corrosion rate.

**Figure 6:**
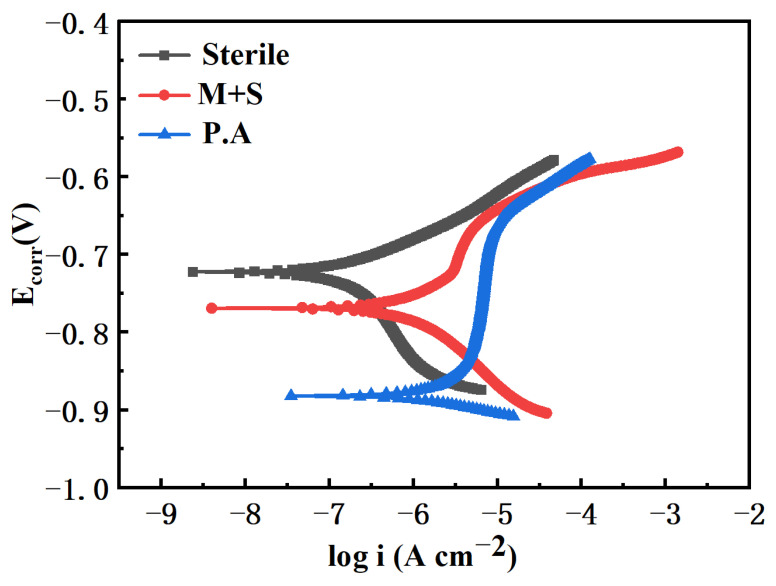
Polarization curves of X65 steel immersed in different medium for 14 days.

Figure 6 shows the polarization curves of X65 pipeline steel in sterile medium, containing wild-type and knockout *P. aeruginosa* after 14 days of immersion. The polarization curves of the knockout *P. aeruginosa* system showed an overall decrease in anodic current, which was lower than that of the wild type. This indicates that the corrosion rate of the knockout *P. aeruginosa* system is smaller, which further suggests that the knockout of the *phzM* and *phzS* genes attenuates the corrosion of X65 pipeline steel by *P. aeruginosa*. Meanwhile, the anodic current of the polarization curve of the knockout *P. aeruginosa* system was also reduced compared with that of the sterile medium, which was attributed to the presence of corrosion product biofilm protecting the steel surface and reducing the corrosion rate.

**Figure 7:**
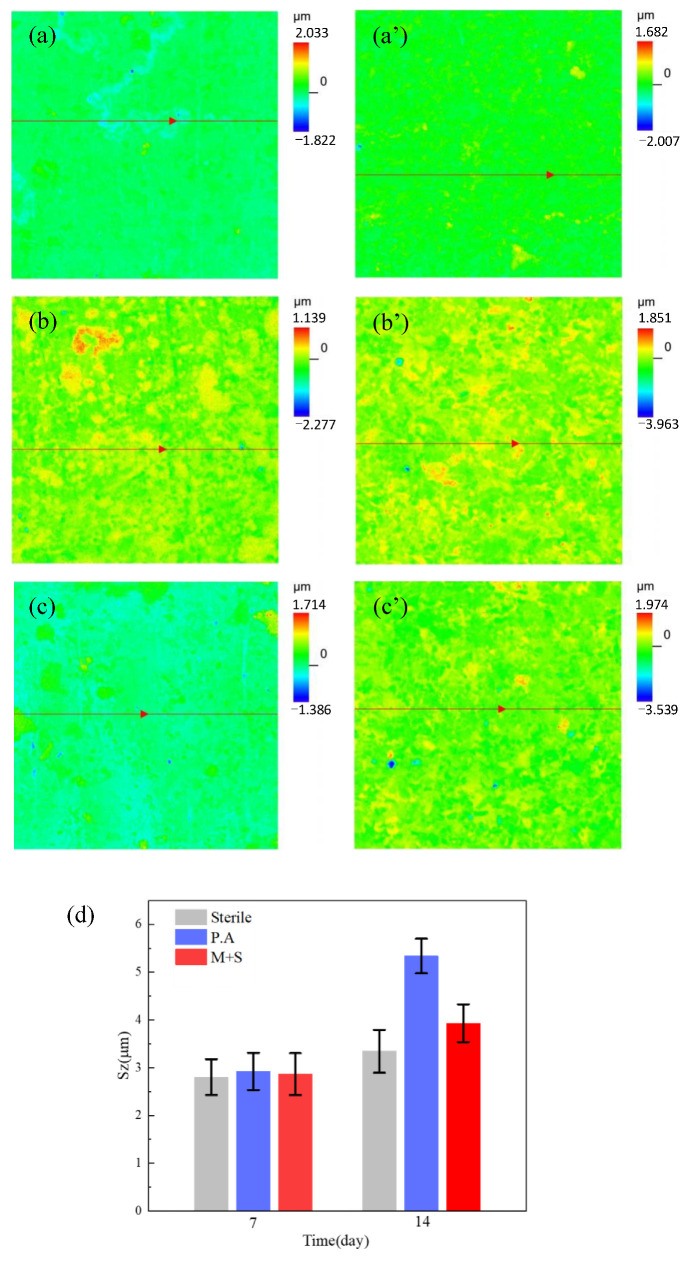
CLSM images of X65 soaked in sterile medium for 7, 14 days (**a**,**a′**), wild-type medium for 7, 14 days (**b**,**b′**) and knockout medium for 7, 14 days (**c**,**c′**) and roughness comparison (**d**). The red lines mark the location of the line scan results of depth in subfigure (**a**–**c′**).

In order to quantitatively characterize the roughness of the mild steel surface after 7 and 14 days of immersion in the corresponding experimental medium, the image of the surface is shown in Figure 7a–d. The roughness parameter calculated over the entire measured surface is Rz—the maximum vertical peak and valley height measurement between the highest and lowest points of the profile. The images and data clearly show that at day 7, the steel surface roughness was more uniform across the three conditions, being 2.81 μm and 2.86 μm in the sterile and knockout medium, respectively, and only 2.92 μm in the wild-type medium, which is slightly higher than the remaining two medium. After the 14th day of immersion, the steel surface roughness reached 3.35 μm and 3.93 μm in the sterile and knockout medium, respectively, while the steel surface roughness in the wild-type medium was 5.34 μm, which was twice as much as that in the sterile medium, and the steel surface was corroded even more severely, which further corroborated the electrochemical results.

### 3.4. pH Analysis of Medium

**Figure 8:**
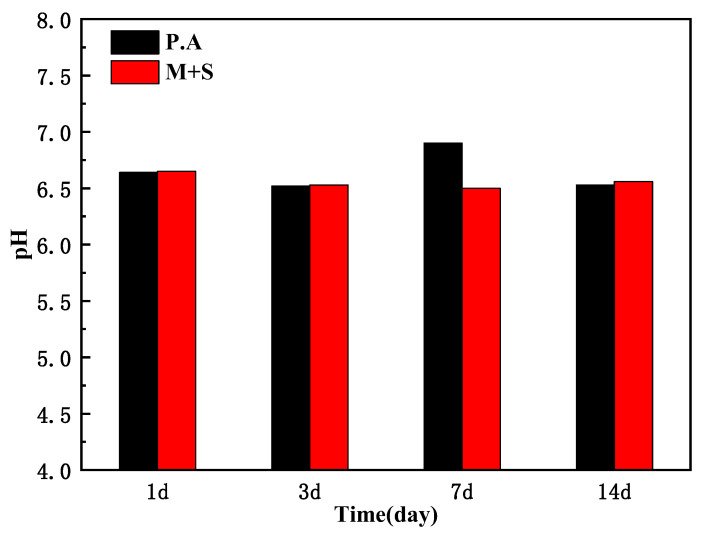
pH of the medium for inoculation of wild-type *P. aeruginosa* and knockout *P. aeruginosa*.

Figure 8 shows the pH values of X65 pipeline steel after 1, 3, 7, and 14 days of immersion in inoculated wild-type and knockout *P. aeruginosa* medium under anaerobic conditions. As shown, *P. aeruginosa* produced acidic metabolites due to anaerobic respiration under anaerobic conditions [24], which caused corrosion of X65 steel, and the pH of both wild-type and knockout *P. aeruginosa* medium was maintained at around 6.5, which demonstrated that knockout of the *phzM* and *phzS* genes did not change the pH of the medium.

## 4. Conclusions

In this paper, the effects of microbial corrosion of wild-type *P. aeruginosa* and knockout *P. aeruginosa* were investigated by taking X65 pipeline steel as the research object, and the conclusions are as follows:

1. From the morphology of the biofilm, the biofilm morphology of the knockout *P. aeruginosa* was consistent with that produced by the wild-type *P. aeruginosa*, indicating that the knockout of the *phzM* and *phzS* genes did not have any particular effect on the biofilm produced by *P. aeruginosa* on the surface of X65 pipeline steel.
2. According to the weight loss and electrochemical results, the weight loss in the wild medium was nearly 8 times greater than that in the knockout environment, and the R*_ct_* value decreased from 1.7 × 10^2^ ± 8.9 Ω∙cm^2^ to 3.8 × 10^4^ ± 2.4 × 10^4^ Ω∙cm^2^ after 14 days of immersion. The corrosion of X65 pipeline steel by *P. aeruginosa* after knockdown of the *phzM* and *phzS* genes was attenuated, suggesting that the *phzM* and *phzS* genes, which regulate the synthesis of PYO, play an important role in accelerating the microbial corrosion of *P. aeruginosa*.

Based on the above conclusions, knocking out specific genes to mitigate the corrosion of Pseudomonas aeruginosa on X65 pipeline steel may have some potential applications, especially in the field of ocean engineering and pipeline transportation. The pipeline materials can be targeted to improve their corrosion resistance. Understanding the corrosive effects of microorganisms on pipelines can help develop more effective pipeline maintenance and management strategies. By monitoring changes in microbial communities and the evolution of corrosion mechanisms, timely measures can be taken to prevent pipeline corrosion and extend the service life of pipelines. This has important application prospects.

## Figures and Tables

**Table 1:** The chemical composition of X65 steel (wt.%).

Element	Mn	Mo	Si	Cr	Cu	Nb	C	V	Ti	Al	Ni	Fe
X65	1.56	0.39	0.23	0.18	0.16	0.05	0.1	0.03	0.03	0.03	0.01	Bal.

**Table 2:** EIS parameters of X65 steel in sterile, wild-type *P. aeruginosa* and gene knockout *P. aeruginosa* medium.

Duration/Day	*R_s_* (Ω∙cm^2^)	*Q_f_* (Ω^−1^ cm^−2^ s^n^)	*R_f_* (Ω∙cm^2^)	*Q_dl_* (Ω^−1^ cm^−2^ s^n^)	*R_ct_* (Ω∙cm^2^)
Sterile					
1	6.7 ± 0.2	1.8 × 10^−5^ ± 3.9 × 10^−6^	1.8 × 10^3^ ± 0.6 × 10^2^	5.5 × 10^−4^ ± 1.3 × 10^−5^	7.5 × 10^3^ ± 3.6 × 10^3^
3	6.7 ± 0.3	4.2 × 10^−5^ ± 3.7 × 10^−6^	2.5 × 10^3^ ± 0.4 × 10^2^	4.5 × 10^−4^ ± 2.6 × 10^−5^	8.3 × 10^4^ ± 1.0 × 10^2^
7	6.4 ± 0.8	1.8 × 10^−4^ ± 5.1 × 10^−5^	2.8 × 10^3^ ± 8.2 × 10^2^	3.1 × 10^−5^ ± 2.8 × 10^−6^	7.1 × 10^4^ ± 1.1 × 10^2^
14	6.4 ± 0.6	1.9 × 10^−4^ ± 5.2 × 10^−5^	3.6 × 10^3^ ± 5.3 × 10^2^	2.9 × 10^−5^ ± 8.9 × 10^−6^	6.2 × 10^4^ ± 8.3 × 10^2^
*P. aeruginosa*					
1	5.9 ± 0.2	4.4 × 10^−5^ ± 1.2 × 10^−5^	1.8 × 10^3^ ± 2.4 × 10^2^	6.9 × 10^−4^ ± 1.6 × 10^−5^	3.1 × 10^2^ ± 2.1 × 10^1^
3	5.6 ± 0.4	7.4 × 10^−5^ ± 2.1 × 10^−6^	2.1 × 10^3^ ± 1.3 × 10^2^	8.7 × 10^−4^ ± 2.2 × 10^−5^	3.5 × 10^2^ ± 8.3 × 10^0^
7	6.0 ± 0.2	7.4 × 10^−5^ ± 8.1 × 10^−6^	2.2 × 10^3^ ± 3.3 × 10^2^	1.3 × 10^−3^ ± 9.3 × 10^−6^	1.9 × 10^2^ ± 2.4 × 10^0^
14	5.6 ± 0.2	1.2 × 10^−4^ ± 5.6 × 10^−5^	2.9 × 10^3^ ± 2.0 × 10^2^	1.6 × 10^−3^ ± 1.4 × 10^−5^	1.7 × 10^2^ ± 8.9 × 10^0^
M+S					
1	4.5 ± 3.9	2.8 × 10^−1^ ± 4.9 × 10^−1^	3.9 × 10^2^ ± 3.4 × 10^2^	3.3 × 10^−1^ ± 5.8 × 10^−1^	2.2 × 10^4^ ± 1.4 × 10^3^
3	6.5 ± 1.2 × 10^−1^	1.1 × 10^−4^ ± 2.1 × 10^−6^	1.2 × 10^3^ ± 4.9 × 10^0^	1.8 × 10^−5^ ± 9.3 × 10^−8^	3.5 × 10^4^ ± 3.1 × 10^3^
7	6.7 ± 1.5 × 10^−1^	1.2 × 10^−4^ ± 2.1 × 10^−6^	5.9 × 10^2^ ± 5.7 × 10^0^	1.6 × 10^−5^ ± 2.1 × 10^−7^	3.8 × 10^4^ ± 4.3 × 10^3^
14	6.4 ± 1.1 × 10^−1^	2.2 × 10^0^ ± 3.8 × 10^0^	1.8 × 10^2^ ± 1.5 × 10^2^	8.9 × 10^1^ ± 1.6 × 10^2^	3.8 × 10^4^ ± 2.4 × 10^4^

## Data Availability

Data are contained within the article.
